# Complete genome sequence of white sturgeon herpesvirus 2 isolated from farmed white sturgeon (*Acipenser transmontanus*)

**DOI:** 10.1128/mra.00420-24

**Published:** 2024-09-30

**Authors:** Andor Doszpoly, Kuttichantran Subramaniam, Karen Kerr, Andrew J. Davison, Thomas B. Waltzek

**Affiliations:** 1 HUN-REN Veterinary Medical Research Institute, Budapest, Hungary; 2 Department of Infectious Diseases and Immunology, College of Veterinary Medicine, University of Florida, Gainesville, Florida, USA; 3 MRC-University of Glasgow Centre for Virus Research, Glasgow, United Kingdom; 4 Washington Animal Disease Diagnostic Laboratory and Department of Veterinary Microbiology and Pathology, Washington State University, College of Veterinary Medicine, Pullman, Washington, USA; Katholieke Universiteit Leuven, Leuven, Belgium

**Keywords:** white sturgeon herpesvirus 2, *Alloherpesviridae*, *Acipenser transmontanus*, sturgeon, genome

## Abstract

The complete genome sequence of white sturgeon herpesvirus 2 (strain UC Davis) was determined. Comparative genomic analyses confirmed the classification of this virus in the species *Ictavirus acipenseridallo2* in the family *Alloherpesviridae*.

## ANNOUNCEMENT

White sturgeon herpesvirus 2 (WSHV2) was first isolated (strain UC Davis) on white sturgeon spleen cells from the ovarian fluid of a white sturgeon in California, USA, during routine sampling of broodstock in 1991 (1). Subsequently, WSHV2 was isolated from wild white sturgeon from Idaho, Oregon, and, again, California (2–4). Previous studies on partial WSHV2 genome sequences utilizing 12 core genes conserved in members of the family *Alloherpesviridae* demonstrated that WSHV2 is a member of the genus *Ictavirus* and distantly related to white sturgeon herpesvirus 1 and lake sturgeon herpesvirus (5–7). As a result, WSHV2 was classified in the species *Acipenserid herpesvirus 2*, which has recently been renamed *Ictavirus acipenseridallo2* (https://ictv.global/home).

WSHV2 (strain UC Davis) virions were purified by centrifugation from infected cell culture medium, and DNA was isolated using phenol:chloroform:isoamyl alcohol and ethanol precipitation, as described previously (5). A sequencing library was prepared using a Nextera XT kit (Illumina) and sequenced on a MiSeq instrument (Illumina) using a v3 600-cycle reagent kit, yielding 1,745,120 paired-end reads. These data were analyzed using default options except where stated otherwise. The untrimmed reads were assembled into a large contig (131,939 bp) using SPAdes v3.10.1 (8) with the --careful, --cov-cutoff auto, and -k 21,33,55,77,99,127 parameters. BLASTX (9) searches against the NCBI non-redundant protein database showed that this contig is related to alloherpesvirus genomes. Contig integrity was verified by trimming the reads using Trim Galore v. 0.6.6 (https://www.bioinformatics.babraham.ac.uk/projects/trim_galore/) with the --illumina and --paired parameters, aligning the trimmed reads to the contig using Bowtie 2 v.2.4.2 (10) and Samtools v.1.12 (11), and inspecting the alignment using Tablet v.1.21.02.08 (12). The genome termini were identified from two large subsets of reads, each consisting of >1,000 reads commencing at the same nucleotide. Two regions containing complex reiterations were resolved by sequencing PCR products (Table 1). Alignment of trimmed reads to the final genome sequence incorporated 96% of reads at an average coverage depth of 2,897 reads/nt.

**TABLE 1:** PCR primers used to resolve regions containing complex reiterations in the WSHV2 genome

Region^*a*^	Forward primer^*b*^	Location^*c*^	Reverse primer^*b*^	Location^*c*^	PCR product^*d*^
A	CTGGGAGTCAGGGTACGTAGTCCA	3579–3602	TATGTGAGCCAATATGTGCTGGGA	5129–5106	1,551
B	TCAGTTGCCGGCAACACTCAAGTG	118942–118965	AGTTCATCCAAGTCTATGTCTCCA	119565–119542	624
B	GACACTATCAATCTTACATCTGAC	119438–119461	TTGAACACCGGGTGGGCCAATGTC	119791–119768	354
B	GACACTATCAATCTTACATCTGAC	119438–119461	CAACTGTGGTTGCGTCTGACGGAA	119849–119826	412
B	GACACTATCAATCTTACATCTGAC	119438–119461	CACTGGGCATGATAAGATCATCAC	119921–119898	484
B	TGGAGACATAGACTTGGATGAACT	119542–119565	CAACTGTGGTTGCGTCTGACGGAA	119849–119826	308
B	TGGAGACATAGACTTGGATGAACT	119542–119565	CACTGGGCATGATAAGATCATCAC	119921–119898	380

^*a*^ Genome region A is located in TR and is therefore present twice in the genome. Genome region B is located in U and is therefore present once in the genome.

^*b*^ Sequences of forward and reverse primers (Sigma-Aldrich) are listed in 5′–3′ orientation in relation to the genome and antigenome sequences, respectively.

^*c*^ Primer locations are listed in relation to the genome sequence. For region A, locations in the copy of TR at the left genome end are listed.

^*d*^ PCR products (nt) are listed acccording to size in the genome sequence. They were produced using a KAPA HiFi Hot Start Readymix PCR kit (Roche Diagnostics) following the manufacturer’s instructions and sequenced (Source Bioscience) on capillary instruments (Applied Biosystems) using the primers that generated them.

**Fig 1:**
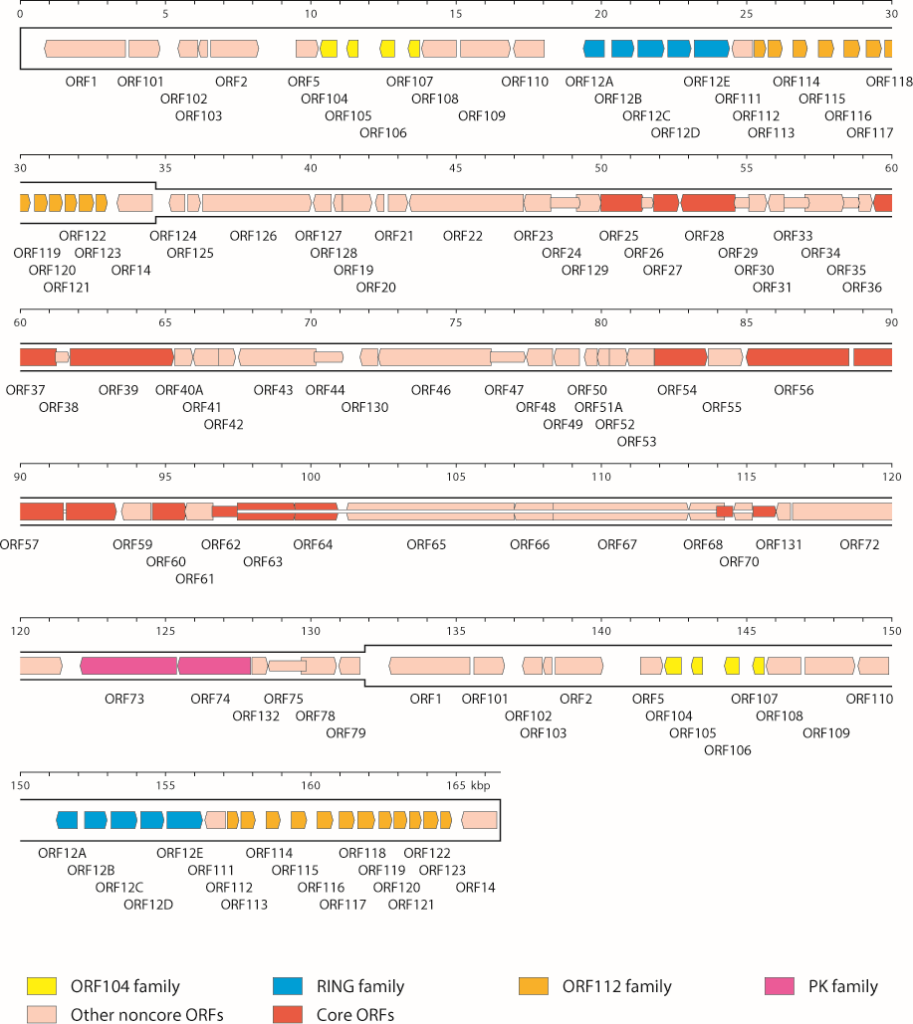
WSHV2 genome map. The unique region is shown in a thinner format than the terminal direct repeats. Predicted functional ORFs are named to correspond to orthologs in other ictaviruses (ORF1–ORF79) or in a separate series (ORF101–ORF132). They are indicated by arrows colored according to the key as belonging to gene families (sets of paralogous genes in the ORF104, RING, ORF112, and PK families), core ORFs (conserved among alloherpesviruses), and other noncore ORFs. Some ORFs are shown by narrow arrows to make their locations clearer. Introns connecting ORFs are shown as narrow white bars.

The linear WSHV2 genome (166,523 bp; 42% G + C) is similar in structure to the genomes of other ictaviruses (13), consisting of a unique region (U; 97,195 bp) flanked by terminal direct repeats (TR; 34,664 bp). Using approaches described previously (6), 127 open reading frames (ORFs) encoding functional proteins were predicted (Fig. 1). Comparative phylogenetic analyses based on the 12 alloherpesvirus core genes confirmed the classification of this virus in the species *Ictavirus acipenseridallo2* (5–7).

This is the first report of a complete genome sequence of WSHV2. Sequences of various WSHV2 strains isolated from white sturgeon available in GenBank are highly similar to the corresponding regions of this sequence (>99% identical using BLASTN; coverage 39%–100%). These include a substantial sequence of 66 kbp (14) and four ostensibly complete genome sequences of 134 kbp (15) that are incomplete due to apparent misidentification of the termini. Short sequences available in GenBank from related viruses isolated from shortnose sturgeon (*Acipenser brevirostrum*) (2) and Siberian sturgeon (*Acipenser baerii*) (16) are less similar to WSHV2 (82%–93% identical; coverage <1%–4%).

## Data Availability

The WSHV2 genome sequence is available in GenBank under accession number PP622675. The sequence reads are available under BioProject accession number PRJNA1098059.
